# 

**Published:** 1869-07

**Authors:** 


### Our Exchanges.

ZELL'S ENCYCLOPEDIA.—The fifth monthly number of this work has been received. We have examined it carefully to see whether it was worthy of a recommendation to our readers, and do not hesitate to say that it is really a work of great value. It is indeed a "universal dictionary," and contains every information that can be obtained from Gazetteers, Dictionaries of Biography, Medicine, Law, Languages, and the Sciences, all complete in two volumes.

It is handsomely printed on good paper, and beautifully and profusely illustrated. It is sold so cheap, that every family can afford to have one. Send $1.00 for the first ten numbers, and you will be sure to purchase them all. T Elwood Zell. No. 17 and 19 South Sixth-st. Philadelphia, Pa.

PETERSON.—The great magazine for Ladies. Really the cheapest ladies' Magazine in the world. You get all manner of information from it, suited to every housekeeper. It tells you how to cut and make your own and children's dresses. How to embroider and crochet—how to make bead, shell and hair work. Gives you receipts for cooking for the well and sick—all for $2,00 pr year. Address Chas. J. Peterson, 306 Chestnut street, Philadelphia Pa.

THE NORTHERN PENNSYLVANIAN.—A real live, wide-a-wake weekly published at Great Bend, Pa. Its editor, L. H. Whittlesey, is altogether the spiciest "local" anywhere in this neighborhood. It is a treat to read his paper *through.* He doubtless has a large circulation for it, as he *ought* to have.

YOUNG FOLK'S FRIEND—Is the title of a useful little journal published at Sparta, Ga., N. Drahcir is its Editor, and it is afforded monthly at the low price of fifty cents. We advise our young folks to subscribe for the "Friend," as you not only secure a real chatty, lively, little paper, but also a useful "premium packet" free to every subscriber. Address "R. A. Harrison, Publisher Young Folk's Friend," Sparta, Ga.

PETERS' MUSICAL MONTHLY.—Wonder whether our musical friends have seen this beautiful magazine. It is chuck full of the best music published.

For $3.00 per annum, you can secure all of the best songs, chorusses, and music for the piano, that would cost ten times as much if purchased in sheet form. Send thirty cents to J. Peters, 198 Broadway, New York, and secure a specimen copy.

"THE UNIVERSE"—Is the title of a new, large, neatly printed, quarto weekly eight page paper, consolidated from the "Chicagoan," "Sorosis" and "Advance Guard." It is devoted to the "elevation and independence of Woman," contains a health department for the benefit of the house-wife, and has secured Robert Dale Owen and Epes Sargent as regular writers for it columns. It is a real, live, wide-a-wake journal that should be taken in every intelligent family. Terms $2,50 per year, or will be sent on trial three months for fifty cents. Address "The Universe," Chicago, Ill.

THE PRACTICAL PAINTER.—Is a new and neat little journal, published by Wills, Macdonald & Co., at 37 Park Row, New York, at fifty cents per year. Every Artist and Painter should subscribe for it, as it contains much valuable information of paints and painters.

### NEW BOOKS.

POLLARD'S BOOK.—"The Life of Jefferson Davis with a Secret History of the Southern Confederacy, gathered behind the Scenes in Richmond," is the title of a new book by Edward A Pollard.

We have received advance sheets of this work and find it to be a well written and intensely interesting books. Mr. Pollard edited one of the Richmond journals during the war, and was, of course, admitted to many of the secret conclaves of the men who managed affairs in that rebel city. His criticisms of Mr. Davis' management of the Confederate Government are severe, and place that gentleman in an entirely new light before the American People. From this book we learn who were responsible for the success and failures of the Confederacy, and who their really great men were.

We are sure the book will be of great interest to men both North and South, and that it will meet with a ready and great sale.

It will be sold by subscription only, and Agents are wanted in every county. Address :—

"National Publishing Company,"
26 South 7th St. Philadelphia, Pa.

---

HOUSEKEEPER'S DEPARTMENT is unavoidably crowded out—we are sorry—"Housekeeper" made her department a valuable one. We will give it place next time.

### Answers to Correspondents.

Under this head we will answer all communications addressed to us of interest to the general reader, and will reply to such persons who forget to enclose us a postage stamp.

We will cheerfully give our advice in this department to all who seek it, and solicit correspondence upon all subjects of interest to the people generally.

Mrs. D.—*Sunbury, Pa.*—Six months is old enough for your child to have an operation for Club-Foot.

E. B.—*Huntingdon Pa.*—Your friend must come for examination. Know nothing of the physician you mention.

Rev. C. C.—*Sardinia, N. Y.*—No safe treatment can be recommended for your friend unless under the care of some good physician.

Dr. J. W.—*Cario. Ill.*—"Stellwag on the Eye"—Published by Wm. Wood & Co., N. Y., is the best work on the eye published in this country.

Mrs. J.—*Williamsport, Pa.*—"Babcock's Uterine Supporter," is the very best in use. The price is $25, and can be had of Dr. L. A. Babcock, Freeport, Ill.

L. M,—*Olney, Ill.*—We do not care to advertise the different trusses in use, gratuitously. Write us enclosing stamp, and we will tell you which we deem the best.

T.—*Waverly, N. Y.*—Quit the use of quack medicines and apply to some of your village physicians for relief. Dr. Johnston of your place will be a good man to employ in such a case.

E. S.—*Ponca, Nebraska.*—You will see a treatnent for rhumatism in this number of the BISTOURY, under the head of "Medical Items and News." Show it to your family physician, who can carry out the treatment.

Dr. S.—*Moravia, N Y.*—You will find the treatment for your case, in the "Medical Items and News." 2nd, The Medical Record of N. Y., Wm. Wood & Co. Publishers, price $4 per year, is the best journal in this state.

A. H. S.—*Greenwood, N. Y.*—Thanks for subscription and kind words. Can't you send us a Club from your place? Try, please.

The physician you speak of is *not* of the "regular faculty.

Dr. J.—*Syracuse, N. Y.*—"A Treatise on Diseases of the Ear," by Anton Von Troltsch, M. D., is the title of the work. Send to Wm. Wood & Co., New York.

Geo. Tiemann & Co., New York, can furnish you with the eye instrument.

Dr. C,—*Madison, Wis.*—Will answer your queries at length in October No. of BISTOURY, when they will *fit* the season.

In the meantime, had'nt you better subscribe for BISTOURY? You may fail to get it without.

Mrs. R.—*Hornellsville, N. Y.*—Procure an ounce of *Solution of Carbolic Acid.* Place from 10 to 20 drops in a pint of warm water, agitate well with the syringe, then apply it every evening with a bulb syringe. You will find it a most excellent application for ulcerated uterus.

S. R. C.—*Marietta, Pa.*—Inquires about the Elmira Water Cure.

Of course its a good Institution or we would not recommend it in the BISTOURY.

No better place, of the kind, in this country for people with chronic diseases.

Mrs. M. A. L.—*Oakland, Cal.*—Your eye difficulty depends upon some disturbance of your system. Cannot tell what from your discription. Better consult an Oculist near you.

Forwarded BISTOURY to names sent. Hope they will become subscribers.

Mrs. J. B.—*Honesdale, Pa.*—The next time your "Cancer Doctor" appears with his cancer plaster, broomstick him from the house. He will not only rob you of your money, but of your *life*, as well. Apply to some surgeon and have your breast removed with the knife, and so save yourself a world of pain and trouble.

J. T.—*Dansville, N. Y.*—Do not apply "sugar of lead" to your eyes—it is a very dangerous application. Rather use a collyrium made of common table salt twenty grains; rose-water, one ounce, wine of opium, half a drachm. Apply this twice every day. If you have nothing more dangerous than simple inflammation of the eye, it will give you relief.

G. R.—*Rochester, N. Y.*—Read the last number of the BISTOURY for our opinion of the parties you mention. It is *always* better to trust your family physician with the treatment of such a difficulty, rather than to employ those who agree to send you formulæ *free of charge.* Every honorable physician will sympathise with you and keep your secret.

R. M. M.—*Shasta Valley, Cal.*—After complimenting the BISTOURY very highly, asks for a prescription for palpitation of the heart, giving other symptoms of his case too long to insert here.

Procure one once of the *Bromide of Potassium.* Place it in one pint of water, and, when dissolved, take a teaspoonful every night just before retiring. Report the result in two months.

S. B. I.—*Lawrenceville, Pa.*—Has *ozenæ*, a formation of tenacious scabs in the nose which are very offensive. Wants to know what he can do to'rd getting relief.

Procure a nasal douche—then run a pint of warm water, in which you have placed fifteen drops of the *solution of Carbolic Acid*, through one nostrel and out the other, every morning and evening. This will give you relief, and *may*, in time, cure you.

Dr. (?) B.—*Adrian, Mich.*—Wants to sell us a recipe for a "cancer plaster," "warranted to cure the worst cancer that ever was," Wants to know what we will give him for it. We answer:—

Give you "fits," should we ever hear of your applying your "remedy" to anyone. Or, if you prefer it, we'll give five cents, if you agree to hang yourself upon receipt of it.

We have seen too much misery caused by such applications as this fellow proposes to dispose of to have any patience whatever with those who deal in them.

J. T. H.—*Vernon, Hill Va.*—Complains of sore, tender feet, during the warm months, and wants to know how to relieve them.

Bathe your feet in warm water every night—rub them perfectly dry with a crash towel, then rub well with a liniment composed of Alcohol, one pint; Tincture of Camphor. three ounces, Liquor Amonia, one ounce.

Follow up this treatment every night for two or three months, and the tenderness will leave your feet. Hope you will send a Club from your place.—See terms on inside of cover.

